# A Synchronized Two-Dimensional $\alpha $–$\Omega $ Model of the Solar Dynamo

**DOI:** 10.1007/s11207-023-02173-y

**Published:** 2023-07-17

**Authors:** M. Klevs, F. Stefani, L. Jouve

**Affiliations:** 1 Helmholtz-Zentrum Dresden – Rossendorf, Bautzner Landstr. 400, D-01328 Dresden, Germany; 2 Institute for Numerical Modelling, University of Latvia, 3 Jelgavas street, Riga, LV-1004 Latvia; 3 Univ. Toulouse, IRAP, CNRS, UMR 5277, CNES, UPS, F-31400 Toulouse, France

**Keywords:** Solar cycle, Models, Helicity, Theory

## Abstract

We consider a conventional $\alpha $–$\Omega $-dynamo model with meridional circulation that exhibits typical features of the solar dynamo, including a Hale-cycle period of around 20 years and a reasonable shape of the butterfly diagram. With regard to recent ideas of a tidal synchronization of the solar cycle, we complement this model by an additional time-periodic $\alpha $-term that is localized in the tachocline region. It is shown that amplitudes of some decimeters per second are sufficient for this $\alpha $-term to become capable of entraining the underlying dynamo. We argue that such amplitudes of $\alpha $ may indeed be realistic, since velocities in the range of m s^−1^ are reachable, e.g., for tidally excited magneto–Rossby waves.

**Supplementary Information:**

The online version contains supplementary material available at 10.1007/s11207-023-02173-y.

## Introduction

The general idea that solar-activity variations might be linked to the orbital motion of the planets traces back to Wolf (1859), and it was kept alive, throughout one and a half centuries, by a number of authors (de la Rue, Stewart, and Loewy, 1872; Bollinger, 1952; Jose, 1965; Takahashi, 1968; Wood, 1972; Öpik, 1972; Condon and Schmidt, 1975; Charvatova, 1997; Zaqarashvili, 1997; Landscheidt, 1999; Palus et al., 2000; De Jager and Versteegh, 2005; Wolff and Patrone, 2010; Abreu et al., 2012; Callebaut, de Jager, and Duhau, 2012). The more specific coincidence, though, of the 11.07-year alignment cycle of the tidally dominant planets Venus, Earth, and Jupiter with the Schwabe cycle was brought to the fore only recently by Hung (2007), Scafetta (2012), Wilson (2013), and Okhlopkov (2016).

Since even such a remarkable agreement between the average values of two periods might still be a pure coincidence, the question of whether there is a phase coherence between the two time series becomes of the utmost importance. The possible phase stability of the Schwabe cycle was first discussed in the article “Is there a chronometer hidden deep in the Sun?” by Dicke (1978). Analyzing the ratio between the mean square of the *residuals* (i.e. the distances between the instants of the actual cycle maxima and the hypothetical maxima according to a linear trend) to the mean square of the differences between two consecutive residuals, Dicke’s conclusions favored a clocked process over a random walk process. However, apart from the poor statistics connected with the mere 25 maxima taken into account, one should also take seriously Hoyng’s later warning (Hoyng, 1996) that any $\alpha $-quenching mechanism could easily lead to a sort of self-stabilization of the solar dynamo, making a genuine random walk process “disguise” itself as a clocked process – at least for some centuries. A complementary type of cycle stability appears as a typical feature of conventional Babcock–Leighton dynamos, whose period is largely determined by the turnover time of the meridional circulation (Dikpati and Charbonneau, 1999; Charbonneau and Dikpati, 2000; Charbonneau, 2020), which is indeed assumed to be much less fluctuating than the $\alpha $-effect in the convection zone.

With those caveats in mind, we recently re-considered (Stefani et al., 2020b) the longer time series of cycle minima/maxima as bequeathed to us by Schove (1983), and matched them with two series of the cosmogenic isotopes ^10^Be and ^14^C. Apart from the possible existence, or not, of two “lost cycles” (or phase jumps) around 1563 (Link, 1978) and 1795 (Usoskin, Mursula, and Kovaltsov, 2002), our analysis confirmed, by and large, Dicke’s conclusion in favor of a clocked cycle, now throughout the last millennium. More recently, still, this conclusion was contested both by Nataf (2022), who recalled the customary objection against Schove’s data as being “finagled” by his “nine-per-century” rule (Usoskin, 2017), as well as by Weisshaar, Cameron, and Schüssler (2023), who derived – from the new ^14^C data of Brehm et al. (2021) and Usoskin et al. (2021) – a Dicke ratio apparently pointing to a random walk rather than to a clocked process. Yet, the latter result was in turn criticized by Stefani, Beer, and Weier (2023) who identified in the minima/maxima data of Usoskin et al. (2021), as adapted by Weisshaar, Cameron, and Schüssler (2023), a false additional cycle around 1845, and one further additional cycle amidst the Maunder minimum with a similarly low plausibility. The cancellation of both “superfluous” cycles was shown to re-establish the one-to-one correspondence with Schove’s series and thereby the phase stability of the solar dynamo back to 1140 (at least). Keeping the additional cycle around 1650 in place would just imply the existence of one single phase jump within the Maunder minimum, which is by no means in contradiction to the general synchronization concept.

While this entire controversy about solar-dynamo synchronization in the last millennium is still ongoing, it should also be put into the context of the most remarkable, although widely overlooked, work of Vos et al. (2004), whose analysis of two series of algae-related data from 10,000 – 9000 cal. BP (calibrated years before the present) had demonstrated a phase-stable Schwabe cycle with a period of 11.04 years.

In view of those two independent thousand-year long segments, showing nearly identical Schwabe cycles with periods between 11.04 and 11.07 years (which are, within the error margins of the respective data, barely distinguishable), and the strong evidence for phase stability in either case, we consider it at least worthwhile to seek a possible physical mechanism that could be capable of linking the weak tidal forces, as exerted by planets, with the solar dynamo. Setting out from the numerical observation (Weber et al., 2013, 2015; Stefani et al., 2016) that a tide-like influence (with its typical $m=2$ azimuthal dependence) can entrain the *helicity oscillation*^1^ of an underlying $m=1$ instability (the Tayler instability (Tayler, 1973; Seilmayer et al., 2012), for that matter), with barely changing its energy content, we have pursued some rudimentary synchronization studies in the framework of simple 0D and 1D $\alpha $–$\Omega $-dynamo models (Stefani et al., 2016, 2017, 2018; Stefani, Giesecke, and Weier, 2019). Within the same framework, we recently tried (Stefani et al., 2020a; Stefani, Stepanov, and Weier, 2021) to explain also the longer term Suess–de Vries cycle in terms of a beat period (Wilson, 2013; Solheim, 2013) between the fundamental 22.14-year Hale cycle and the 19.86-year period of the Sun’s barycentric motion (forced, in turn, by the orbits of Jupiter and Saturn (Cionco and Pavlov, 2018)). With the intervening spin–orbit coupling remaining poorly understood, we resorted to the same buoyancy-instability mechanism as had been employed by Abreu et al. (2012) to explain typical modulation periods on the centennial time-scale. Yet, this similarity between the final results notwithstanding, the fundamental time-scales of our model (22.14 and 19.86 years) that generate the much longer beat period of 193 years, are still close to the period of the undisturbed dynamo. Our mechanism for explaining long-term modulations might, therefore, be less vulnerable to stochastic noise than what was discussed by Charbonneau (2022) in relation to the original model of Abreu et al. (2012).

Admittedly, being restricted to the latitudinal coordinate, our simple 1D dynamo model did not have the requisite level of detail to give a quantitative answer to Charbonneau’s recent question of “what, then, can be considered a physically reasonable amplitude for external forcing” (Charbonneau, 2022). It was all the more encouraging that, utilizing a 2D Babcock–Leighton model with a periodic perturbation of the lower operating-field threshold of the source term, Charbonneau (2022) found a similarly robust synchronization mechanism as Stefani, Giesecke, and Weier (2019). Such a variation of the lower operating-field threshold would correspond to variations of the field-loss parameter $\kappa $ as employed by Stefani et al. (2020a) and Stefani, Stepanov, and Weier (2021) to parameterize the spin–orbit coupling with its 19.86-year periodicity. While we do not exclude a viable physical translation of the (11.07-year periodic) tidal forcing into such a type of variation of the field-storage capacity, in this article we will stick to our original idea that it is essentially the $\alpha $-effect that is affected by the tides. Specifically, we seek to know then *how much of this periodic* $\alpha $*-variation would be needed* to accomplish synchronization of an otherwise conventional $\alpha $–$\Omega $-dynamo. Guided by a rough estimation based on the equipartition assumption $U_{\mathrm{pot}}\approx E_{\mathrm{kin}}$, we consider approximately 1 m s^−1^ as an upper limit for the tide-induced velocity variation. Given that the value of $\alpha $, which reflects only the helical part of the turbulence, is typically one order of magnitude lower than the underlying velocity, the focus of our modelling will be on whether $\alpha $-values on the order of dm s^−1^ are sufficient to entrain the entire solar dynamo.

To answer this specific question, we step back from the more sophisticated double-synchronization model of Stefani et al. (2020a) and Stefani, Stepanov, and Weier (2021) and restrict ourselves to the very basic tidal synchronization of the Schwabe/Hale cycle. In the next section, we present a rather conventional two-dimensional $\alpha $–$\Omega $-dynamo with meridional circulation ${\boldsymbol {u}}_{\mathrm{p}}$, utilizing observation-constrained values for $\Omega $ and ${\boldsymbol {u}}_{\mathrm{p}}$, and employing more or less realistic values of $\alpha $ and the magnetic diffusivity $\eta $. To keep the model simple, no specific Babcock–Leighton source term is added to the $\alpha $-effect “living” in the convection zone. In the next section, we first adjust the value of $\eta $ to provide a reasonable natural period of the undisturbed dynamo. While the most simple form of the $\alpha $–$\Omega $ model leads, as usual, to a badly shaped butterfly diagram (with dominating poleward migration), the correct butterfly shape is recovered by switching on the meridional circulation. Based on the reference model thus defined, we will then assess in detail how much $\alpha $-variation in the tachocline region is actually needed for synchronization.

The article will conclude with a short discussion of the results and some prospects for future work.

## The Model

In this section, we motivate and describe our mean-field solar dynamo model and discuss its numerical implementation. Considering only axi-symmetric solutions, we work with a system of partial differential equations whose spatial variables are the co-latitude and the radius. Intentionally, the model has been kept similarly simple as the benchmark model of Jouve et al. (2008).

As usual, the magnetic field is split into a poloidal component ${\boldsymbol {B}}_{\mathrm{P}}(r,\Theta ,t)=\nabla \times (A(r,\Theta ,t) { \boldsymbol {e}}_{\phi})$ and a toroidal component ${\boldsymbol {B}}_{\mathrm{T}}(r,\Theta ,t)=B(r,\Theta ,t) {\boldsymbol {e}}_{\phi}$. The main sources of dynamo action are the gradient of the angular velocity $\Omega $ and the $\alpha $-effect resulting from the helical part of the turbulence in the convection zone. While our model is not a Babcock–Leighton model (which would require a particular source term at the surface) it is a flux-transport model, since it comprises a meridional circulation ${\boldsymbol {u}}_{\mathrm{p}}$, mainly to ensure a realistic shape of the butterfly diagram.

Choosing the solar radius ${\mathrm{R}}_{\odot}=695{,}700\text{ km}$ as the length and the diffusive time ${\mathrm{R}}_{\odot}^{2}/\eta _{t}$ as the time scale, we employ here – as in Jouve et al. (2008) – the dimensionless form of the coupled induction equations for the azimuthal components $B \equiv B_{\phi}$ of the magnetic field and $A \equiv A_{\phi}$ of the vector potential,

1 $$\begin{aligned} \frac{{\partial} B}{{\partial} t} =& \tilde{\eta} D^{2} B+ \frac{1}{s}\frac{\partial (s B)}{\partial r} \frac{\partial \tilde{\eta}}{\partial r} - R_{\mathrm{m}} s {\boldsymbol {u}}_{\mathrm{p}} \cdot \nabla \left ( \frac{B}{s} \right ) +C_{\Omega} s (\nabla \times (A{\boldsymbol {e}}_{\phi}))\cdot \nabla \Omega\;, \end{aligned}$$

2 $$\begin{aligned} \frac{{\partial} A }{{\partial} t} =&\tilde{\eta} D^{2} A- \frac{R_{\mathrm{m}}}{\mathrm{s}}{\boldsymbol {u}}_{\mathrm{p}} \cdot \nabla (s A)+ C^{\mathrm{c}}_{ \alpha} \alpha ^{\mathrm{c}} B + C^{\mathrm{p}}_{\alpha} \alpha ^{\mathrm{p}} B\;, \end{aligned}$$

wherein we use the notations $D^{2} \equiv (\nabla ^{2}-s^{-2})$, $s \equiv r \sin \theta $, and $\tilde{\eta}=\eta /\eta _{\mathrm{t}}$, with $\eta _{\mathrm{t}}$ being the turbulent magnetic diffusivity in the convection zone.

This system is governed by four magnetic Reynolds numbers characterizing, respectively, the effects of shear, meridional circulation, and two different $\alpha $-terms:

3 $$\begin{aligned} C_{\Omega} =&\Omega _{\mathrm{eq}} {\mathrm{R}}^{2}_{\odot} /\eta _{\mathrm{t}}\;, \end{aligned}$$

4 $$\begin{aligned} R_{\mathrm{m}} =&u_{0} {\mathrm{R}}_{\odot}/ \eta _{\mathrm{t}}\;, \end{aligned}$$

5 $$\begin{aligned} C^{\mathrm{c}}_{\alpha} =&\alpha ^{\mathrm{c}}_{\mathrm{max}} {\mathrm{R}}_{\odot} /\eta _{ \mathrm{t}}\;, \end{aligned}$$

6 $$\begin{aligned} C^{\mathrm{p}}_{\alpha} =&\alpha ^{\mathrm{p}}_{\mathrm{max}} {\mathrm{R}}_{\odot} /\eta _{ \mathrm{t}} \;. \end{aligned}$$

Herein, $\Omega _{\mathrm{eq}}=2 \pi \times 456$ nHz is the angular velocity at the Equator, and $u_{0}$ and $\alpha ^{\mathrm{c}}_{\mathrm{max}}$ and $\alpha ^{\mathrm{p}}_{\mathrm{max}}$ are the typical intensities of the meridional circulation and the two separate $\alpha $-effects in the convection zone and in the tachocline region. In contrast to Guerrero and de Gouveia Dal Pino (2007), Jouve et al. (2008), and Sanchez et al. (2014), we do not incorporate any specific Babcock–Leighton source term.

We suppose the turbulent magnetic diffusivity [$\eta _{\mathrm{t}}$] in the convection zone to be dominated by a strong $\beta $-effect, whereas it is much smaller in the relatively quiet tachocline region. Refraining from more complicated structures of $\eta $ as employed, e.g., by Guerrero and de Gouveia Dal Pino (2007) or Sanchez et al. (2014), we use here the simple form of Jouve et al. (2008)

7 $$\begin{aligned} \tilde{\eta}(r) =& \frac{\eta _{\mathrm{c}}}{\eta _{\mathrm{t}}} +\frac{1}{2} \left (1- \frac{\eta _{\mathrm{c}}}{\eta _{\mathrm{t}}}\right ) \left [1 +{\mathrm{erf}} \left (\frac{r-r_{\mathrm{c}}}{d}\right ) \right ] \end{aligned}$$

with $\eta _{\mathrm{c}}=0.01 \eta _{\mathrm{t}}$, $r_{\mathrm{c}}=0.7$, and $d=0.02$, which shows a smoothed-out jump (by a factor of 100) between the radiation zone and the convection zone.

**Figure 1:**
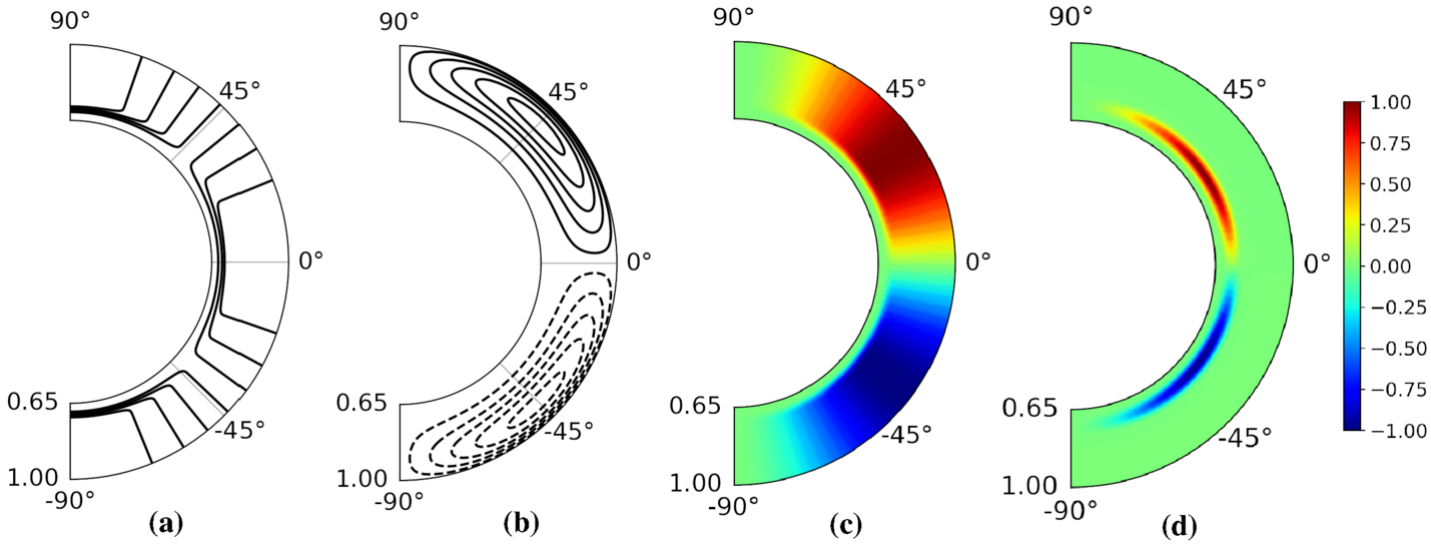
Spatial structures of the main ingredients of the dynamo model in the meridional plane. **(a)** Isolines of $\Omega (r,\Theta )/\Omega _{\mathrm{max}}$. **(b)** Streamlines of ${\boldsymbol {u}}_{\mathrm{p}}(r,\Theta )$. **(c)** Constant part of $\alpha $, taken in the unquenched state: $\alpha ^{\mathrm{c}}(r,\Theta )/\alpha ^{\mathrm{c}}_{\mathrm{max}}$. **(d)** Periodic part of $\alpha $, with the resonance term set to 1: $\alpha ^{\mathrm{p}}(r,\Theta )/\alpha ^{\mathrm{p}}_{\mathrm{max}}$.

For the angular velocity, we apply the same spatial structure as Jouve et al. (2008):

8 $$\begin{aligned} {\Omega}(r,\Theta ) =&C_{\Omega} \left \{ \Omega _{\mathrm{c}}+\frac{1}{2} \left [ 1 +{\mathrm{erf}} \left ( \frac{r-r_{\mathrm{c}}}{d} \right ) \right ] (1- \Omega _{\mathrm{c}}-c_{2} \cos ^{2} \Theta ) \right \} \end{aligned}$$

with $r_{\mathrm{c}}=0.7$, $d=0.02$, $\Omega _{\mathrm{c}}=0.92$, and $c_{2}=0.2$ (see Figure 1a).

For the meridional circulation we chose, again as in Jouve et al. (2008), one single cell defined by ${\boldsymbol {u}}_{\mathrm{p}}= \nabla \times (\psi (r,\Theta ) {\boldsymbol {e}}_{\phi})$ with the stream function

9 $$\begin{aligned} \psi (r,\Theta ) =& R_{\mathrm{m}} \left \{ -\frac{2}{\pi} \frac{(r-r_{\mathrm{b}})^{2}}{(1-r_{\mathrm{b}})} \sin \left ( \pi \frac{r-r_{\mathrm{b}}}{1-r_{\mathrm{b}}} \right ) \cos \Theta \sin \Theta \right \} \end{aligned}$$

with $r_{\mathrm{b}}=0.65$ (see Figure 1b). We are well aware of the fact that the specific structure of ${\boldsymbol {u}}_{\mathrm{p}}$ is much less settled than that of ${\Omega}(r,\Theta )$, and that more complicated two-cell flows (Kosovichev et al., 2022) might also be considered in future improvements of our model.

Finally, $\alpha =\alpha ^{\mathrm{c}}+\alpha ^{\mathrm{p}}$ is thought to consist of a conventional part $\alpha ^{\mathrm{c}}$ in the convection zone, whose time-dependence stems only from the quenching by the magnetic field,

10 $$\begin{aligned} \alpha ^{\mathrm{c}}(r,\Theta ,t) =&C^{\mathrm{c}}_{\alpha} \frac{3 \sqrt{3}}{4}\sin ^{2} \Theta \cos \Theta \left [1+{\mathrm{erf}} \left ( \frac{r-r_{\mathrm{c}}}{d} \right ) \right ]\left [1+ \frac{|{\boldsymbol {B}}(r,\Theta ,t)|^{2}}{B^{2}_{0}} \right ]^{-1} \end{aligned}$$

with $B_{0}=1$, and an explicitly time-dependent (with forcing period $T_{\mathrm{f}}$) part $\alpha ^{\mathrm{p}}$ that is concentrated in the tachocline region,

11 $$\begin{aligned} \alpha ^{\mathrm{p}}(r,\Theta ,t) =&C^{\mathrm{p}}_{\alpha} \frac{1}{\sqrt{2}} \sin ^{2} \Theta \cos \Theta \left [1+{\mathrm{erf}} \left ( \frac{r-r_{\mathrm{c}}}{d} \right ) \right ] \left [1-{\mathrm{erf}} \left ( \frac{r-r_{\mathrm{d}}}{d} \right ) \right ] \times \\ & \frac{2|{\boldsymbol {B}}(r,\Theta ,t)|^{2}}{1+|{\boldsymbol {B}}(r,\Theta ,t)|^{4}} \sin (2 \pi t/T_{\mathrm{f}} )\;, \end{aligned}$$

where $r_{\mathrm{d}}=0.75$. Note that the factor on the second line of Equation 11 represents a resonance term as introduced by Stefani et al. (2016) in order to account for a field-dependent optimal reaction of the underlying instability (e.g. Tayler instability) on the tidal forcing. A similar field dependence has been used, e.g., by Charbonneau (2022), although with the slightly different interpretation as a nonlinearity of the non-local source term that incorporates both a lower and upper operating threshold for the strength of the toroidal magnetic field at the base of the convection zone. The spatial structures of these two $\alpha $-terms are presented in Figure 1c, d, in either case disregarding any magnetic-field dependence.

For the numerical solution, an explicit finite-difference scheme in two dimensions in spherical coordinates is used, partly with the standard resolution of $64 \times 64$ grid points in radial and latitudinal direction (as originally used by Rüdiger, Elstner, and Ossendrijver (2003)), partly with an enhanced resolution of $128 \times 128$. The equations are solved with perfect-conductor boundary conditions $A=\partial (r B)/ \partial r=0$ at $r=0.65 {\mathrm{R}}_{\odot}$ and vertical field conditions $B_{\phi}=B_{\Theta}=0$ at $r={\mathrm{R}}_{\odot}$.

## Results

In this section, we present and assess the results of three dynamo models with increasing complexity.

### Non-synchronized Model, Without Meridional Circulation

**Figure 2:**
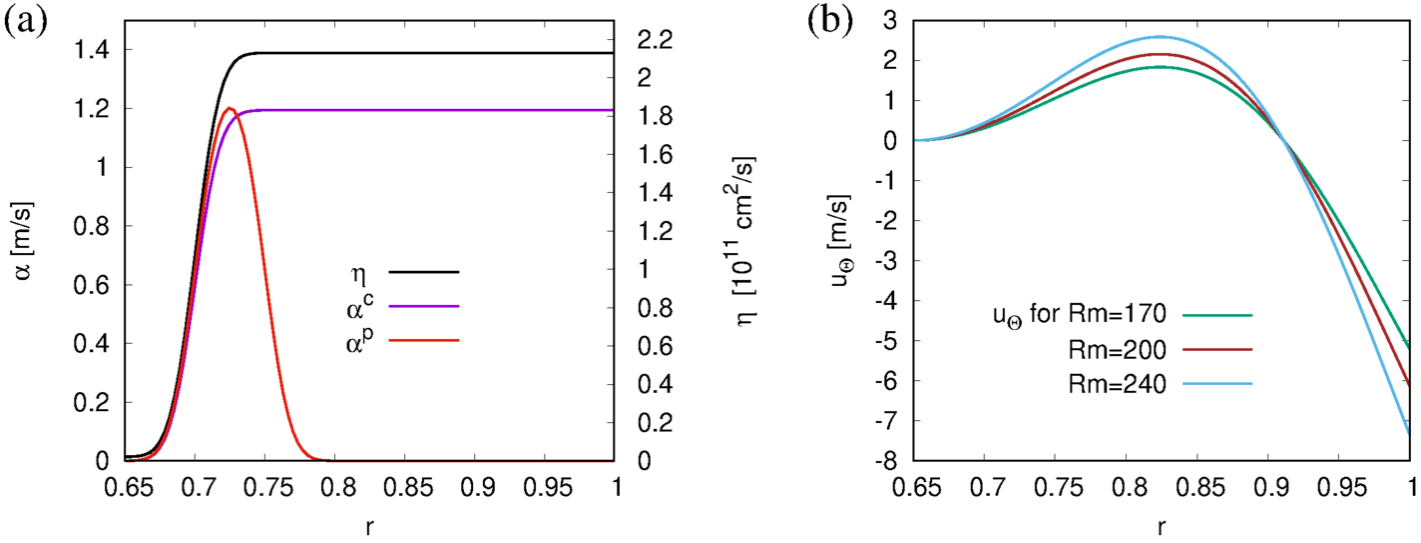
Radial dependence of various dynamo ingredients in physical units, all taken at $\Theta =45^{\circ}$. **(a)** Diffusivity $\eta (r)$ (*black*), $\alpha ^{c}(r)$ in the unquenched form (*violet*), and $\alpha ^{p}(r)$ for $\alpha ^{p}_{\mathrm{max}}=\alpha ^{c}_{\mathrm{max}}$ and with the field-dependent resonance factor artificially set to 1 (*red*). **(b)** $u_{\Theta}(r)$ resulting from the stream function of Equation 9 for three different $R_{m}$.

First, we consider the simplest case of Parker’s migratory dynamo (Parker, 1955), without any synchronization term ($\alpha ^{\mathrm{p}}=0$), and without meridional circulation (${\boldsymbol {u}}_{\mathrm{p}}=0$). For the sake of concreteness, we set $\eta _{\mathrm{t}}=2.13 \times 10^{11}\text{ cm}^{2}\,\text{s}^{-1}$, and $\alpha ^{\mathrm{c}}_{\mathrm{max}}=1.30\text{ m}\,\text{s}^{-1}$, both of which are close to the respective geometric mean of the lower and upper values, as typically found in the literature ($10^{10}$ – $10^{13}\text{ cm}^{2}\,\text{s}^{-1}$ for $\eta $ and 10 – $10^{3}\text{ cm}\,\text{s}^{-1}$ for $\alpha $, see Charbonneau (2020)). The resulting magnetic Reynolds numbers according to Equations 3 and 5 are $C_{\Omega}=65{,}100$ and $C^{c}_{\alpha}=42.46$. The radial dependencies of $\eta (r)$ and $\alpha ^{\mathrm{c}}$ (in its unquenched form) are illustrated, for $\Theta =45^{\circ}$, in Figure 2a. Note that for this particular angle, $\alpha ^{\mathrm{c}}(r)$ does not reach the maximum value of $1.30\text{ m}\,\text{s}^{-1}$.

**Figure 3:**
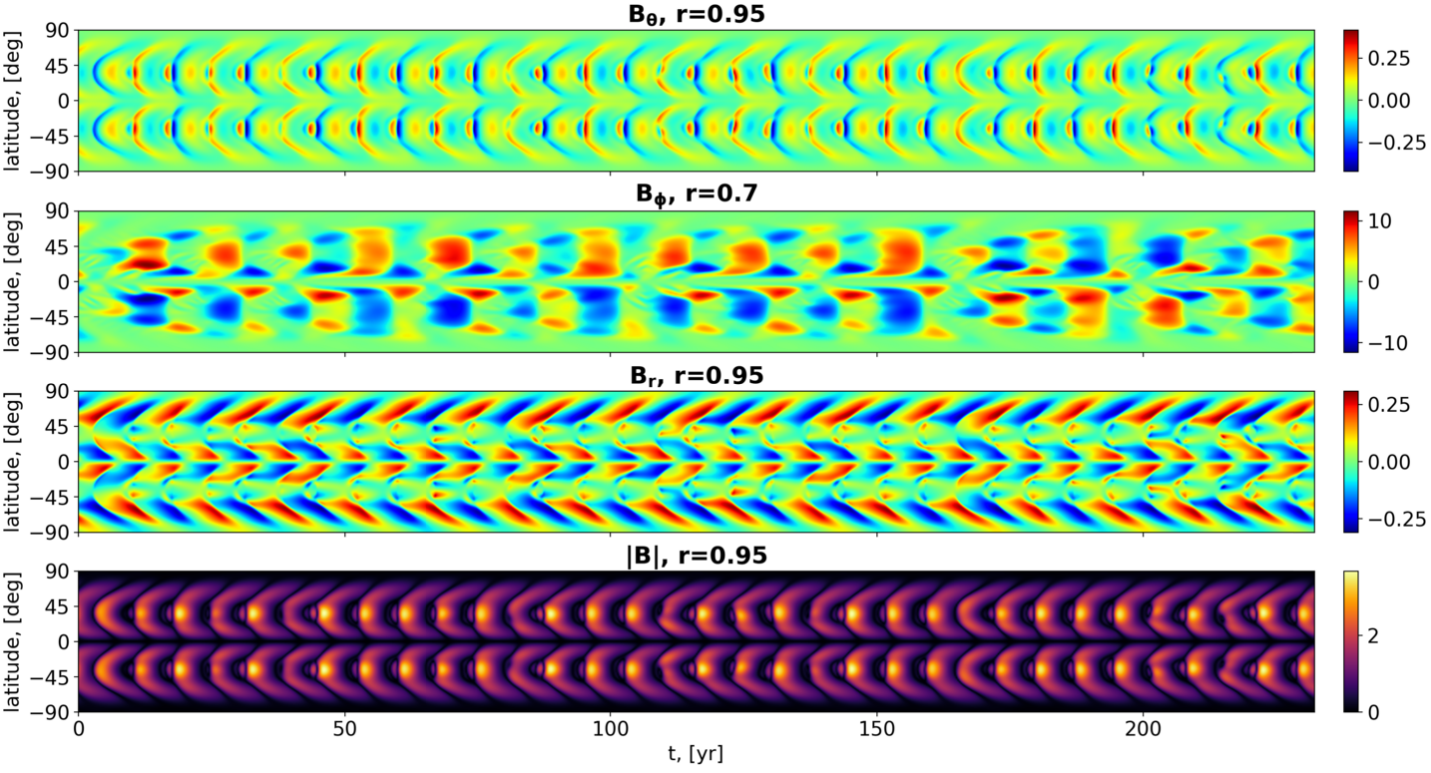
Contour-plots of $B_{\Theta}(r=0.95,\theta ,t)$, $B_{\phi}(r=0.7,\theta ,t)$, and $B_{r}(r=0.95,\theta ,t)$ and of $|{\boldsymbol {B}}(r=0.95,\theta ,t)|$ for the non-synchronized model without meridional circulation. The simulations were carried out with the enhanced resolution of $128 \times 128$. Note that the ordinate axis represents not the colatitude $\theta $, but the normal solar latitude $90^{\circ}-\theta $.

Figure 3 illustrates the resulting field dependence on time and latitude, taken partly at $r=0.95$, partly at $r=0.7$, showing a reasonable dynamo-cycle period of $T_{\mathrm{d}}=14.27$ years (i.e. 0.0198 diffusion times), but a badly shaped butterfly diagram.

### Non-synchronized Model, with Meridional Circulation

In order to recover the correct shape of the butterfly diagram, we switch on a meridional circulation, setting its value to $u_{0}=5.2\text{ m}\,\text{s}^{-1}$, which corresponds to $R_{\mathrm{m}}=170$. For this value, as well as for $R_{\mathrm{m}}=200$ and 240, the radial dependence of $u_{\Theta}$ is shown, again for $\Theta =45^{\circ}$, in Figure 2b. While the values $u_{\Theta}$ at $r=1$ are by a factor of approximately two too low compared with observations, the typical values of 1 – 2 m s^−1^ at the base of the convection zone are quite compatible with values from helioseismology. Actually, the latter velocities are the crucial ones to set the cycle period.

**Figure 4:**
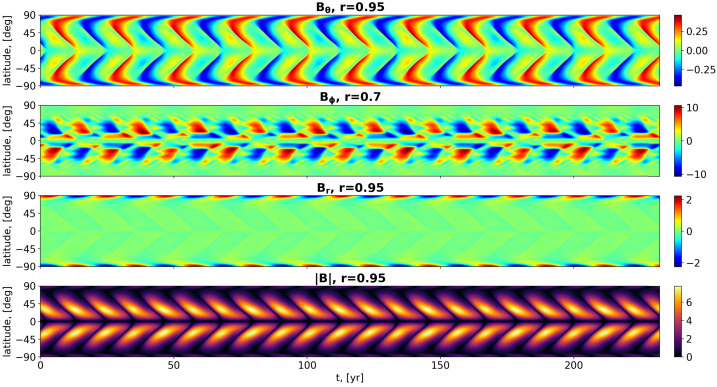
Contour-plots of $B_{\Theta}(r=0.95,\theta ,t)$, $B_{\phi}(r=0.7,\theta ,t)$, and $B_{r}(r=0.95,\theta ,t)$ and of $|{\boldsymbol {B}}(r=0.95,\theta ,t)|$ for the non-synchronized model including meridional circulation with $R_{\mathrm{m}}=170$. The simulations were carried out with the enhanced resolution of $128 \times 128$. Note that the ordinate axis represents not the colatitude $\theta $, but the normal solar latitude $90^{\circ}-\theta $.

As seen in Figure 4, we obtain now a butterfly diagram of rather decent shape and a slightly changed cycle period of $T_{\mathrm{d}}=22.798$ years. This will serve in the following as the reference dynamo model, whose synchronization is to be evaluated thereupon. While further improvements of the spatio-temporal features of the magnetic field are certainly possible (for example, when including an appropriate Babcock–Leighton source term), we refrain from any further sophistication of the model.

### Synchronized Model

Finally, we switch on the periodic $\alpha $-term with an assumed forcing period of $T_{f}=11.00$ years (we do not insist here on the precise value of 11.07 years). The radial dependence of $\alpha ^{\mathrm{p}}$ is illustrated by the red curve in Figure 2a. Note, however, that here $\alpha ^{\mathrm{p}}_{\mathrm{max}}$ has the same value of $1.30\text{ m}\,\text{s}^{-1}$ as the corresponding $\alpha ^{\mathrm{c}}_{\mathrm{max}}$, and that the field-dependent resonance term in Equation 11 is set to its maximum value of 1, which is reduced outside the optimal magnetic-field value.

**Figure 5:**
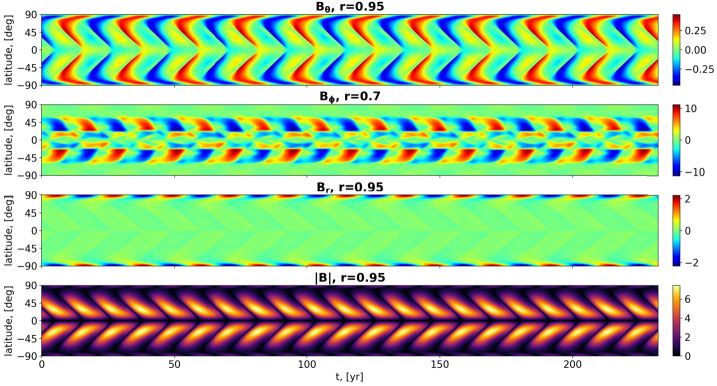
Contour-plots of $B_{\Theta}(r=0.95,\theta ,t)$, $B_{\phi}(r=0.7,\theta ,t)$, and $B_{r}(r=0.95,\theta ,t)$ and of $|{\boldsymbol {B}}(r=0.95,\theta ,t)|$ for the synchronized model including meridional circulation with $R_{\mathrm{m}}=170$ and a periodic $\alpha $-term with amplitude $\alpha ^{\mathrm{p}}_{\mathrm{max}}=0.52\text{ m}\,\text{s}^{-1}$ and period $T_{\mathrm{f}}=11.00$ years. The simulations were carried out with the enhanced resolution of $128 \times 128$. Note that the ordinate axis represents not the colatitude $\theta $, but the normal solar latitude $90^{\circ}-\theta $.

As shown in Figure 5, for the specific value $\alpha ^{\mathrm{p}}_{\mathrm{max}}=0.52\text{ m}\,\text{s}^{-1}$ we obtain now the dynamo period $T_{\mathrm{d}}=22.00$ years, which corresponds to twice the period $T_{\mathrm{f}}$ of the forcing. Apart from that, there is barely any significant change in the field structures compared with the non-synchronized case in Figure 4. A video illustrating the field dynamics in the synchronized case can be found in the Electronic Supplementary Material.

**Figure 6:**
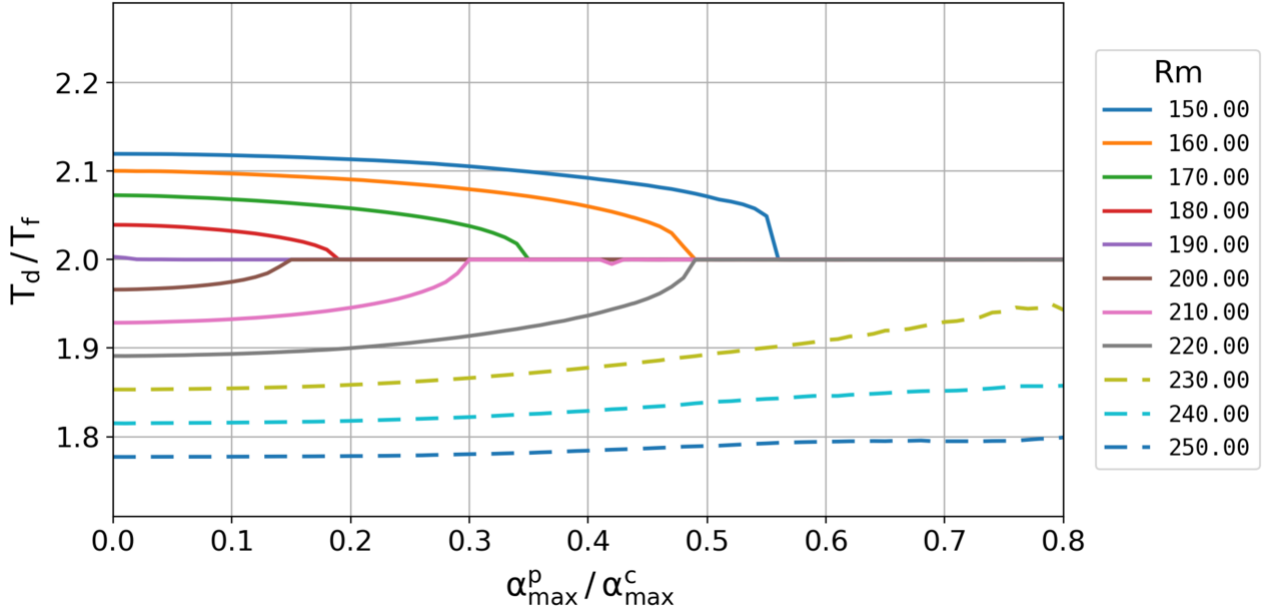
Ratio of the period $T_{\mathrm{d}}$ of the signal to the period $T_{\mathrm{f}}$ of the forcing dependence on the relative strength of the forcing $\alpha ^{\mathrm{p}}_{\mathrm{max}}/\alpha ^{\mathrm{c}}_{\max}$. The color-coded curves refer to different ratios of the “natural” period $T_{\mathrm{n}}$ of the non-synchronized dynamo to $T_{\mathrm{f}}$, which has been varied by changing the magnetic Reynolds number $R_{\mathrm{m}}$ of the meridional circulation. $T_{\mathrm{n}}$ can be read from the value on the ordinate axis multiplied by 11 years; it amounts, for example, to 23.3 years for $R_{\mathrm{m}}=150$, to 21.6 years for $R_{\mathrm{m}}=200$, and to 19.5 years for $R_{\mathrm{m}}=250$. These numerically expensive simulations were carried out with the standard resolution of $64 \times 64$.

In Figure 6, we present the dependence of the dynamo period $T_{\mathrm{d}}$ on $\alpha ^{\mathrm{p}}_{\mathrm{max}}$. Here we have used a couple of ratios of the “natural” period $T_{\mathrm{n}}$ (of the non-synchronized dynamo with $\alpha ^{\mathrm{p}}_{\mathrm{max }}=0$) to the forcing periods $T_{\mathrm{f}}$ by simply changing the amplitude of meridional circulation, which governs $T_{\mathrm{n}}$. Very similar to Figure 10 in Stefani, Giesecke, and Weier (2019), and to Figure 10 in Charbonneau (2022), we obtain a clear parametric resonance for some critical value of $\alpha ^{\mathrm{p}}_{\mathrm{max}}$ that depends on the initial distance between twice the forcing period $T_{\mathrm{f}}$ and the natural period $T_{\mathrm{n}}$ of the unperturbed dynamo. As we had chosen $\alpha ^{\mathrm{c}}_{\mathrm{max}}=1.30\text{ m}\,\text{s}^{-1}$, synchronization occurs for an amplitude of $\alpha ^{\mathrm{p}}_{\mathrm{max}}$ in the range of some decimeters per second. The relative smallness of this number is, of course, a consequence of the 100 times smaller value of $\eta $ in the tachocline region, which amplifies correspondingly the induction effect of $\alpha ^{\mathrm{p}}$, even if the latter is concentrated in a significantly smaller zone than $\alpha ^{\mathrm{c}}$. That said, we must also admit that synchronization requires a certain proximity of $2 T_{\mathrm{f}}$ and $T_{\mathrm{n}}$; for the $R_{\mathrm{m}}$, values indicated by the dashed lines in Figure 6, no clear synchronization was observed even for the highest considered value of $\alpha ^{\mathrm{p}}_{\mathrm{max}}/\alpha ^{\mathrm{c}}_{\mathrm{max}}=1$. This narrowness of the synchronizability region, which somewhat contrasts with the broader region obtained in the framework of the 1D model (Figure 10 of Stefani, Giesecke, and Weier (2019)), might have to do with the tight scaling of $T_{\mathrm{n}}$ with the period of the meridional circulation.

## Conclusions

As a sequel to the 0D and 1D modelling of solar-cycle synchronization (Stefani et al., 2016, 2018; Stefani, Giesecke, and Weier, 2019; Stefani et al., 2020a; Stefani, Stepanov, and Weier, 2021), we have investigated a more realistic 2D $\alpha $–$\Omega $-dynamo model. Starting from a conventional set-up without meridional circulation, exhibiting a poorly shaped butterfly diagram, via an enhanced model with meridional circulation showing the correct butterfly shape, we have assessed the synchronization capabilities of a time-periodic $\alpha $-term concentrated in the tachocline region. For rather standard values of all other parameters, it was shown that synchronization starts already at a magnitude of this additional $\alpha $-term as low as some decimeters per second. The smallness of this value relies on the fact that $\eta $ in the quiet-tachocline region is significantly lower than in the convection zone, where it is dominated by the turbulent $\beta $-effect. The utilized tachoclinic diffusivity $\eta \approx 2.13 \times 10^{9}\text{ cm}^{2}\,\text{s}^{-1}$ should be considered a conservative choice; in view of much lower values such as $2.2 \times 10^{8}\text{ cm}^{2}\,\text{s}^{-1}$ as used by Guerrero and de Gouveia Dal Pino (2007), the real value of $\alpha $, required for synchronization, might still be lower than the one derived here.

This brings us back to Charbonneau’s “elephant in the room: what, then, can be considered a physically reasonable amplitude for external forcing?” (Charbonneau, 2022). Let us recall the very rough estimation (Öpik, 1972) that the typical tidal height of $h_{\mathrm{tidal}} = {\mathrm{G}} m R^{2}_{\mathrm{tacho}}/(g_{\mathrm{tacho}} d^{3}) \approx 1\text{ mm}$ corresponds energetically to a velocity scale of $v_{0} \approx (2 g_{\mathrm{tacho}} h_{\mathrm{tidal}})^{1/2} \approx 1\text{ m}\,\text{s}^{-1}$ when employing the huge gravity at the tachocline of $g_{\mathrm{tacho}} \approx 500\text{ m}\,\text{s}^{-2}$. Invoking the equally rough estimate $\alpha \approx v_{0}$ from renormalization theory (Moffatt and Dormy, 2019) (and even when realistically assuming $\alpha $ to be one or two orders of magnitude smaller than $v_{0}$), a tidally generated $\alpha $-value of a few decimeters per second seems not out of reach. Indeed, it was recently shown (Horstmann et al., 2023) that (magneto–)Rossby waves (Marquez-Artavia, Jones, and Tobias, 2017; Zaqarashvili, 2018; Dikpati et al., 2020) under the influence of a *realistic* tidal forcing are capable of acquiring velocity scales of up to 1 m s^−1^. Therefore, it appears that the “astrological homeopathy” (Charbonneau, 2022) of tidal forcing may well be suited to generate an $\alpha $-effect in the tachocline region that is strong enough to entrain the entire solar dynamo.

We have further confirmed the prior results of Stefani, Giesecke, and Weier (2019) (Figure 10) and Charbonneau (2022) (Figure 10) that this type of synchronization requires a certain proximity of the tidal forcing’s period to half the “natural” period of the undisturbed dynamo. The Sun, therefore, may just be in the lucky situation of being orbited by a Jupiter with a period that fits nicely to half the “natural” period of the undisturbed dynamo, in contrast to a number of exoplanets for which no sign of synchronization was found by Obridko, Katsova, and Sokoloff (2022). It remains to be seen whether some peculiar features of the solar dynamo, e.g. its somewhat unusual cycle period (Böhm-Vitense, 2007) and, in particular, “its comparatively smooth, regular activity cycle” (Radick et al., 2018), could find an explanation in such a rare case of parametric resonance. At any rate, it should be noted that in our case the relation of the planet’s orbital period to the rotation period of the star is completely different from that of some “hot Jupiters”, exerting a much stronger tidal forcing, for which other types of resonances in the form of spin–orbit commensurabilities were recently discussed by Lanza (2022).

What are the next steps to be taken? First and foremost, the specific action of $m=2$ tidal forces on various $m=1$ instabilities (e.g. Tayler) or waves (e.g. magneto–Rossby), and on the $\alpha $-effect connected with them, has to be quantified in a reliable manner. Complementary work on tidal influences on Rayleigh–Bénard convection, and its large-scale circulation (Stepanov and Stefani, 2019; Jüstel et al., 2020, 2022), might be helpful to elucidate helicity entrainment in a more generic sense.

Second, the possible role of further axisymmetric induction effects, beyond the $\alpha $-effect, has to be clarified. The basic idea of a torque-influenced magnetic-buoyancy instability within the tachocline (Ferriz Mas, Schmitt, and Schüssler, 1994; Zhang et al., 2003; Abreu et al., 2012) might play a central role here. It was indeed employed as the basic synchronization mechanism by Charbonneau (2022), while Stefani et al. (2020a) and Stefani, Stepanov, and Weier (2021) had used it only to bring into play the second fundamental period 19.86 years via spin–orbit coupling (yet poorly understood, but see Javaraiah (2003), Shirley (2006), Sharp (2013) for first estimates). It certainly needs much more work to disentangle these two effects. Further to this, we should not overlook alternative axisymmetric ($m=0$) instabilities, the possible relevance of which had been discussed by several authors (Dikpati et al., 2009; Rogers, 2011). The recently discovered helical magnetorotational instability for flows with positive radial shear (Mamatsashvili et al., 2019) might be an particularly interesting candidate in this respect.

## Supplementary Information

Below is the link to the electronic supplementary material.

(MP4 8.9 MB)

## Footnotes

1 While this intrinsic helicity oscillation of the Tayler instability was originally found in the simple setting of a non-rotating, full cylinder, a similar effect was recently observed also in a much more realistic 3D simulation of a stably stratified and rotating tachocline, see Figure 16 of Monteiro et al. (2023).
